# Achieving Smoke-Free Mental Health Services: Lessons from the Past Decade of Implementation Research

**DOI:** 10.3390/ijerph10094224

**Published:** 2013-09-10

**Authors:** Sharon Lawn, Jonathan Campion

**Affiliations:** 1 Department of Psychiatry, Flinders Human Behaviour and Health Research Unit, Flinders University, Room 4T306, Margaret Tobin Centre, Flinders University, GPO Box 2100, Adelaide 5001, Australia; 2 South London and Maudsley NHS Foundation Trust, Bethlem Royal Hospital, Monks Orchard Road, Beckenham PR3 3BX, UK; E-Mail: Jonathan.Campion@slam.nhs.uk; 3 Department of Population Mental Health, University College London, UCL Partners, London WC1E 7HB, UK

**Keywords:** smoke-free policy, mental disorder, mental illness, smoking, smoking culture, mental health services, psychiatric inpatients

## Abstract

The culture of smoking by patients and staff within mental health systems of care has a long and entrenched history. Cigarettes have been used as currency between patients and as a patient management tool by staff. These settings have traditionally been exempt from smoke-free policy because of complex held views about the capacity of people with mental disorder to tolerate such policy whilst they are acutely unwell, with stakeholders’ continuing fierce debate about rights, choice and duty of care. This culture has played a significant role in perpetuating physical, social and economic smoking associated impacts experienced by people with mental disorder who receive care within mental health care settings. The past decade has seen a clear policy shift towards smoke-free mental health settings in several countries. While many services have been successful in implementing this change, many issues remain to be resolved for genuine smoke-free policy in mental health settings to be realized. This literature review draws on evidence from the international published research, including national audits of smoke-free policy implementation in mental health units in Australia and England, in order to synthesise what we know works, why it works, and the remaining barriers to smoke-free policy and how appropriate interventions are provided to people with mental disorder.

## 1. Introduction

Levels of smoking are much higher in people with mental disorders and, while tobacco consumption has reduced in the general population over the last decades, this has not been the case for this population. In the USA, UK and Australia, over 40% of all tobacco is consumed by people with mental disorders [1,2,3,4]. The position is similar in New Zealand and likely so in other countries too [5].

Smoking has been part of the cultural fabric in mental health care for many decades. Within inpatient settings, this has included the supply of cigarettes to patients (for example, every hour on the hour), and purchase of those cigarettes by the hospital where the patient was indigent and could not buy their own cigarettes. Within long-stay inpatient settings, this has also included patients who are smokers receiving discounted board and lodging fees in order to cover the costs of their smoking [6,7]. In previous decades, many psychiatric hospitals also possessed canteens with a tobacco license. There is evidence that both patients and nursing staff, in particular, have begun smoking as a result of exposure to the smoking culture in mental health settings [6]. This has clear legal and occupational health and safety implications for policy makers, services, staff and patients in these settings [8].

In mental health settings, cigarettes have been used as a patient management tool by staff, mediating exchanges and relationships between staff and patients, and between patients [6,9]. Examples of this are staff using control over supply of cigarettes to patients in order to encourage patients to comply with requests such as taking medication, getting dressed, agreeing to speak with the doctor, or to settle their adverse behaviour. Examples of how cigarettes have mediated relationships between patients include the trade, barter and intimidation of some patients by other patients in order to gain cigarettes. For some more vulnerable patients, this has included sexual favours. Such exchanges have been common knowledge held by staff, as part of the culture [6,7,9]. Smoking has also been incorporated into clinical practice and has included control of tobacco supply to manage behavior of patients, and widespread use of smoking as an activity to keep patients occupied and calm, to create rapport or offer comfort and support [6,10,11]. Smoke-free policies are therefore crucial to address these processes that reinforcement of smoking. Smoke-free policies are an important step in promoting smoking cessation in mental health settings [12], particularly inpatient settings where the culture of smoking has been most apparent.

## 2. Experimental Section

For the purposes of this paper, we examined the issues surrounding smoke-free policy in mental health settings in general. Our goal was to summarize past research by drawing from many separate investigations and highlighting important research gaps [13]. We followed Torraco’s advice concerning literature reviews broadly rather than as an integrated or systemic approach to the existing literature on this topic. That is, we provide a summary of how the literature was identified, analyzed, synthesized and reported [14].

The literature for this review was identified through the authors’ immersion in this field over the past decade. This entailed the first author’s leadership of an international review of the topic [15] and the second author’s involvement in a number of articles [16,17,18], a recent review of smoking and mental disorder [12] and production of NICE guidance on smoking cessation in secondary care. This guidance included evidence reviews of effectiveness of smoking cessation in mental health settings as well as barriers and facilitators for smoking cessation intervention in mental health settings [19,20,21,22]. The authors were also involved in two large national audits of smoke-free policy: they designed and conducted a national audit of 99 mental health units in Australia [23,24] and used this to design a similar national audit in England involving 147 mental health units [25]. Literature was also searched via the Medline, PubMed and CINAHL databases. We used the following search terms: “smoking”; “smoke-free policy”; “mental health”; “hospitals, psychiatric”; “inpatients”; “psychiatric department, hospitals”; and, “smoking cessation”. All abstracts were reviewed, relevant papers were read in full and the key issues raised were identified within each paper. Patterns or themes across the literature were then determined as a means of synthesizing the literature. The aim was to gain a sense of what issues have arisen in mental health units that have implemented or attempted to implement smoke-free policy, and how far the sector has progressed with resolving these issues.

Many of the papers reviewed arose from research conducted in Australia, the UK and the USA; however, we have also drawn on research conducted in countries such as Brazil, Canada, Finland, Germany, Japan, New Zealand, Spain, Sweden and Switzerland. Each of the themes arising from this review is reported briefly in our results and discussion to capture the essence of the concerns raised by this previous body of literature. We hope that this focused approach will stimulate further research in these specific areas. For a more systematic review of research, we refer the reader to a series of recent reports written to inform the NICE guidance in this area [19,20,21,22].

A number of key terms are used in this paper and need clarification.

- The term “mental disorder” is used in preference to “mental illness”. It covers the following recognised diagnoses: depression and anxiety (which may also be referred to as “common mental disorder”); schizophrenia and bipolar disorder (which may also be referred to as “severe mental disorder”); and also personality disorder, alcohol use disorder and drug use disorder.
- “Smoke-free” facilities are understood to be those which have an explicit policy banning the consumption of tobacco within the administrative boundaries of the institution in which the facilities are located. However, the literature is inconsistent. This is because many of the older studies from the 1990s and some of the more recent studies from some countries only recently implementing smoke-free policy, for example, talk about smoke-free policy and mean smoking banned from inside inpatient units and within a certain distance from entrances or windows. Some of the more recent literature is also not explicit, so we are not certain about what is meant. Many units are reported to be smoke-free and yet have dedicated areas within hospital grounds or attached to inpatient units (such as adjoined courtyards). Patients and staff in these settings can go there to smoke, either at their leisure of at dedicated times, dependent on local policy variations. Therefore, when we refer to units with “total” smoke-free policies, we mean units where there is no smoking allowed by patients or staff at any time anywhere inside or in the grounds of psychiatric institution [26]. The literature is consistent in stating that, despite a smoke-free hospital policy being in place, this does not preclude those patients and staff who are able to leave the hospital grounds from doing so in order to smoke.
- The term “partial bans” has been used inconsistently also within the literature. In most papers, it means a policy whereby staff can use their discretion to facilitate smoking by some patients. This discretion is usually based on perceived level of agitation and need, while maintaining a general smoke-free stance towards other patients. In other papers, it means a general smoke-free policy but with designated areas within the hospital grounds where people can go to smoke and/or times when smoking is permitted [26].
- Terms such as “compliance” and “enforcement” have also been used ambiguously. This has therefore confused the debates, especially when determining or arguing whether a smoke-free policy has succeeded or failed. We argue that smoke-free policy is a process, not an event. Therefore, compliance does not mean 100% compliance all the time and in all cases. Likewise, enforcement is something that staff need to develop confidence and skill in performing, over time. They will not enforce smoke-free policy effectively in 100% of situations. We argue that success comes with striving towards a smoke-free goal, as this more accurately reflects the reality of implementation processes. This inconsistency in the use of terms across and within different countries and settings needs to be addressed for research and practice change in this area to progress.

## 3. Results and Discussion

Within systems of care for patients with mental disorder, especially where the culture of smoking is dominant, several myths exist about smoking and mental disorder. This includes the belief, by staff, that failure to supply patients with tobacco will lead to increased patient aggression, that patients are not interested in quitting, and that they are unable to quit. This smoking culture also exists in community mental health settings. Critics of smoke-free policies often regard smoking as a “normal” part of receiving treatment for mental disorder. They also express concern that patients’ mental health would deteriorate without access to tobacco [27,28,29]. Our review discusses seven areas of debate within the existing literature on smoke-free policy in mental health inpatient settings. These areas are outlined in Section 3.1 to Section 3.7 below. They are followed by a summary of positive impacts of smoke-free policy in these settings (Section 3.8), barriers that remain to be addressed (Section 3.9), policy enablers (Section 3.10) and a brief discussion of the limitations of our review (Section 3.11).

### 3.1. Smoke-Free Mental Health Settings and Environmental Tobacco Smoke

Many inpatient mental health facilities are smoke-free in Australia and across the western world; however, such smoke-free policy is often limited to buildings and does not include hospital grounds. The purpose of a smoke-free policy in hospital settings is first and foremost about managing the occupational health and safety of staff, patients and visitors within the setting. It is also about managing an addictive substance which is the biggest contributor to chronic disease and death for all patients. To do nothing about it would therefore seem, on its face, absurd in a therapeutic environment that strives to promote health. As a starting point, hospital administrators and staff have a legal and ethical responsibility to prevent people being exposed to tobacco smoke within an enclosed environment. At this level, the policy is not about quitting. It is about containment and addressing the issue of the presence of environmental tobacco smoke which is now recognized as being as harmful as smoking itself for those exposed to it [30,31].

Prior to the introduction of smoke-free policy, many mental health inpatient units had dedicated “smoking rooms” within the building. However, research shows that smoking rooms, and to a lesser extent smoking in outside areas adjacent to buildings, do not eliminate exposure to environmental tobacco smoke. A cross-sectional study by Ballbe, *et al*. [32], to evaluate environmental tobacco smoke in 64 mental health inpatient units in Catalonia, Spain, found that only units with total indoor and outdoor smoking bans had PM_2.5_ (particle) levels below the standard recommended WHO levels of 10 µg/m^3^. Units with more permissive smoking policies had PM_2.5_ levels from environmental tobacco smoke that have harmful health effects. Staff caring for acutely unwell patients may not be able to leave them unsupervised and may need to administer medicine or otherwise engage with the patient while they smoke in these environments. If there is an adverse incident in a smoking room, staff will need to attend. Other patients will socialize with smokers while they smoke as part of a culture in which there is “nothing else to do”. By default, the designated smoking area can become a meeting point, and fellow smokers will also be exposed to environmental tobacco smoke. Any smoking on site is very likely to result in some environmental tobacco exposure. This also applies to smoking residue on patient and staff clothing. A Large Dutch study of psychiatric inpatient units involving 540 treatment staff, 306 attendants/nurses, and 93 patients [33] found that, due to non-compliance, environmental tobacco smoke exposure is quite high when there is a general smoking ban (designated areas option). They concluded that a total smoke-free policy is the only way to fully protect those working in psychiatry from environmental tobacco smoke exposure, mainly because partial smoke-free policies are not sufficiently complied with. Since the implementation of smoke-free policies, many units in Australian and England continue to include the presence of designated smoking areas within hospital grounds and within easy access to their inpatient units.

### 3.2. Partial and Total Smoke-Free Policy

Research demonstrates clearly that partial smoke-free policies are less successful than total smoke-free policies and create additional problems [15,18,22,33,34]. Settings with partial smoke-free policies usually allow smoking in designated smoking areas. A partial smoke-free policy may designate where, when and how patients are permitted to smoke. This would then give preference to some patients over other patients because they are voluntarily admitted, are in an open ward, or because their individual doctor grants them leave to smoke. Staff may believe that partial smoke-free policies are necessary to support policy compliance [22]. However, the most significant problem with partial smoke-free policies is their limited impact on the staff and patient culture of smoking. They also foster carer systems in which appropriate clinical management of nicotine dependence is undermined. Within hospitals where partial smoke-free policies operate, inconsistency of application of smoke-free policy can lead to staff conflict with other staff, staff conflict with patients, and patient conflict with other patients. This is because it raises equity concerns about access to smoking, which can undermine the policy [18]. Consistency of processes within these already busy and complex care environments is critical for promoting harmony within these settings. It may also be effective to provide a stepped approach from partial to complete smoke-free policy in some contexts. We also acknowledge that total smoke-free policies are easier in certain more restrictive settings, but that certain factors are associated with successful implementation regardless of whether the goal is for a total or partial smoke-free policy [15].

### 3.3. Physical Health Comorbidity and Psychosocial Impacts of Smoking

People with mental disorder experience notably worse physical health than the general population. They have two to three times the mortality and morbidity from the leading chronic health conditions such as cardiac and respiratory disease [35,36]. They die between twenty and thirty year sooner than they should [37]. For example, depression and schizophrenia are associated with significant increased mortality from all disease and reduced life expectancy [38,39,40,41,42].

Of interest, Morris *et al*. [43] and Shabab and West [44] have clearly shown that smokers with mental disorder have better long-term mental health when they quit smoking. Smoking is the single largest cause of premature death. It is arguably the most significant contributor to poor physical health, poverty, community exclusion, and so forth, for this patient population [35]. Research demonstrates that continued smoking drives the perpetuation of cycles of instability of symptoms of mental disorder, in addition to the clear perpetuation of health and social inequalities [43,44,45]. This occurs through insidious poverty created through patients’ need to fund their tobacco consumption, often on limited incomes. The cost of tobacco may consume one third to almost one half of some mental health patients’ total income [46]. Consequently, patients with mental disorder may choose tobacco over purchasing adequate food or paying bills on time. Some patients may be pushed to extreme behaviours such as begging and picking up butts to address their addiction [47]. This arguably reinforces stigma, social exclusion and other markers of disadvantage as part of complex social determinants of health [48].

### 3.4. Clinical Management of Addiction and Mental Disorder

Smoking and mental disorders are thought to share a number of clinical and neurobiological relationships [49]. A pervasive belief held by many patients and staff is that smoking is helpful to the person’s management of the symptoms of their mental disorder. Another belief is that smoking is used by them as a means of coping with stress, to ameliorate cognitive problems and side effects of psychiatric medications, and to relieve boredom and loneliness [11,29,47]. These processes form part of the self-medication hypothesis originally proposed by Khantzian [50]. This hypothesis proposes that patients with mental disorder smoke to self-medicate their psychiatric symptoms and psychosocial circumstances. However, this phenomenon is complex and involves a range of issues related to nicotine withdrawal, deprivation of care within a culture that reinforces smoking and does not adequately address addiction or psychosocial needs, and other factors. A longitudinal naturalistic 5-year prospective research by Levander *et al*. [51], of patients with schizophrenia, found no support for a self-medication hypothesis. 

The management of smoking in mental health settings is first and foremost a question of good clinical practice in relation to recognized addictive behaviours and nicotine dependence [52,53,54]. Nicotine withdrawal is a clinical reality in inpatient contexts, even where there is no smoking-free policy. Often, patients are unable to smoke where they like or as often as they need in order to alleviate symptoms of nicotine withdrawal. Periodic provision of access to tobacco, such as hourly provision of cigarettes in the locked ward, is a poor form of control for nicotine withdrawal. It is likely to heighten dependence by placing the patient in a continual state of peaks and troughs of withdrawal. Within such a system of care, inpatient psychiatric care providers can miss or misinterpret nicotine withdrawal for worsening psychiatric symptoms [49]. This implies that nicotine withdrawal and dependence require proactive management, even where smoking is permitted. It also points to the need to understand the nature of nicotine withdrawal and dependence better within these settings. In particular, it suggests the importance of understanding how to effectively use nicotine replacement therapy (NRT) and other interventions, which are now a standard part of the repertoire of strategies, to address smoking in these settings [16,23].

NRT is the first-line pharmacotherapeutic intervention for addressing smokers’ nicotine withdrawal. Staff should be able to clearly explain its use as a means to achieve long-term smoking cessation where the smoker has decided to stop smoking. Alternatively, staff should be able to explain its use as a support for temporary smoking abstinence because of ward requirements (the smoker does not want to stop but cannot smoke during their admission [19]. Distinguishing these aims is important, to encourage use of NRT for cessation in those who have used it for temporary abstinence. For maximum effectiveness, NRT should be used in the correct dose, in combination (for example, patches and inhaler) and with other non-pharmacological interventions [19]. For patient who refuse NRT initially, it should also be offered more than once during an admission because patients may change their mind and reconsider trying NRT once they have been given time to consider it. Provision of appropriate smoking cessation intervention requires skilled staff that understand the problem and apply informed clinical management of nicotine withdrawal and dependence and incorporate that understanding fully into clinical care. This is similar to treatment of withdrawal from other substances such as alcohol and illicit drugs [15,55]. Staff should also appreciate the potential need for and usefulness of longer-term NRT by this population post-discharge from psychiatric inpatient units, as part of the continuum of cessation support [19,56,57,58].

Another concern of mental health staff is that smoke-free mental health settings will damage their therapeutic relationship with patients, increase patient distress and agitation and increase the number of adverse incidents among the patient population. However, this is not borne out by the evidence and staff have generally had more concerns than the patients [15,17,18,21,22,59,60,61,62]. Hempel, *et al*. [63] Australian research demonstrated decreased patient aggression and decreased staff injuries when tobacco was removed from the inpatient psychiatric setting. Quinn’s [64] U.S. research showed a significant drop in aggressive incidents post implementation of the policy, with verbal aggression decreasing by 45% and physical aggression incidents decreasing by 50% (266 to 133). Internationally, most studies of the implementation of smoke-free policy have sought patients’ views prior to and post implementation of the policy. The overwhelming response of patients has been that they were very concerned about the policy beforehand but far less so once the policy was implemented [15,58].

Another concern is that patients’ mental health status will decline as a consequence of smoke-free policy [22]. A U.S. study of the impact of smoke-free policy on psychiatric symptoms of inpatients found a small but significant worsening of Global Assessment of Functioning scores, but no significant changes to Brief Psychiatric Rating Scale scores or cardiometabolic measures when comparing these scores before and after implementation of the policy [65]. A further concern is that units where smoke-free policy is in place will experience more patients who avoid admission [22]. The only published research focused specifically on whether smoke-free policy actually deters patients from seeking admission when unwell is from Canada [66]. It is based on administrative data records from 2002 to 2005 measuring the impact of two smoking cessation policies on emergency department use, one imposed in a specific psychiatric hospital in 2005 and the other across the entire province of Ontario in 2006. Kurdyak himself said, “The CAMH-specific (Canadian Adult Mental Health-specific) smoking cessation policy had no impact on psychiatric emergency department visit rates in any diagnostic category. The province-wide smoking cessation policy resulted in a 15.5% reduction in patient visits for patients with a primary diagnosis of psychotic disorder” [66]. The study has a number of important limitations that could account for this later finding. These include service restructures and policy and practice changes across the broader mental health service landscape during that time. The authors of the paper suggest that community services have an important role to play in ensuring the continuum of communication and care across inpatient and community mental health and primary health services for such patients.

Other clinical concerns are that patients who smoke but are subject to smoke-free policy will seek early discharge against medical advice, that they will require more PRN medication, and that distress caused by nicotine withdrawal will lead to higher rates of seclusion and restraint. A UK study in a medium secure unit found that, although 64% of the staff supported smoke-free policy, 43% reported experiencing patient management issues. These included increased patient verbal aggression and increased use of staff time in supervising patients smoking [67]. However, several studies have found that these concerns are unfounded [15,21,22,62,65,68,69,70]. This suggests that greater success may relate to how staff attitudes are addressed and the comprehensiveness with which clinical management of nicotine withdrawal occurs. A number of studies have found that staff who are current smokers are more likely to hold negative beliefs about smoke-free policy [22,62,67,69]. Therefore, addressing staff smoking, nicotine withdrawal while on duty for those who do not wish to quit, and attitudes held by staff smokers, should also be a particular priority in these settings [15]. This has also been recommended by NICE [22].

### 3.5. Mental Health Patients and Quitting

There is general concern that psychiatric patients might be less psychologically equipped to initiate and sustain a quit attempt. However, smokers with mental disorder are just as interested in quitting as smokers in the general population [71,72,73] and have, in fact, been shown in some studies to be more interested [52]. An Australian study of 97 psychiatric inpatients [74] found that approximately 47% of smokers reported having made at least one quit attempt within the past 12 months, despite nearly three quarters (71.2%) being classified as in a precontemplative stage of change. Also, the perception that patients with mental disorder cannot stop smoking is directly challenged. This is because many patients experience periods of abstinence, forced or otherwise in their day-to-day lives, and many do quit [29,71].

There is evidence that smoking can become the dominant coping style used by smokers, at the expense of developing other ways of coping [75]. This is important because, when people with mental disorder enter smoke-free psychiatric units, it may be the first time many of them have been exposed to NRT. It may also be the first time they have access to trained mental health staff support to quit smoking. This support can involve psychological and practical coping strategies for addressing nicotine withdrawal. 

There is also the argument that smoke-free policy is ineffective because patients subject to inpatient smoke-free policy inevitably resume smoking once discharged. While it is correct that many patients will resume smoking, this overlooks the fact that most smokers make several quit attempts before stopping permanently and that a smoke-free policy is about considerably more than initiating an attempt to quit. A period of abstinence has a direct health and financial benefit for the patient and has been found to change patients’ perception of their need to smoke, thereby increasing the likelihood that they will successfully quit in the future [52,75]. A study in a large Australian forensic psychiatry setting [58] is particularly pertinent because patients there had no access to leave and resided in the unit long-term. Many patients (42%) stated that they wanted to quit when they arrived at the forensic hospital, 39% of patients were angry with being forced to stop smoking and 76% reported difficulty quitting. However, 85% indicated that it was easier to stop when no-one else smoked and many stated that living in a totally smoke-free environment was the only way they felt they could quit. Over half the patients surveyed continued to not smoke post discharge (average 305 days) [58]. Therefore, while many patients initially objected to the policy, for many this was effective at supporting smoking cessation. A large US study found that many patients remained smoke-free long after they were discharged from hospital because their quit process commenced in hospital and was followed through with simple support in the community [76]. Prochaska, *et al*. [77] earlier study found that, while all patients resumed smoking within three months of discharge, 48% of smokers were abstinent for 24 h immediately following discharge and were almost seven times more likely to make a subsequent quit attempt. This suggests that smoking cessation support during the transition between inpatient and community mental health services is vital [22]. 

A Swiss study [78] reported increased tobacco consumption in light and moderate smokers during hospitalization. The study involved surveys with 91 inpatients and 110 staff members in 2001 (before smoke-free policy), and 134 inpatients and 85 staff members in 2005 (once smoke-free policy was introduced) at a Swiss University psychiatric hospital. No significant changes in smoking prevalence or consumption levels were observed, although more patients considered stopping. Daily cigarette consumption after admission changed significantly between 2001 and 2005. A marked decrease in daily cigarette consumption after three days in hospital, compared to the week before entry, was observed in 2005 (*p* = 0.005); whereas, in 2001, the trend was towards increased smoking (*p* = 0.06). A study involving follow-up of 196 patients post-discharge from a smoke-free Brazilian psychiatric hospital [79] found that, while 31% maintained the same level of smoking, 27% had reduced their smoking, 10% had quit and relapsed, and 27% had quit smoking completely. A further study [80] confirmed that patients reported an increased expectancy of success with quitting and a decreased expectancy of difficulty with staying quit, as a result of being in a smoke-free inpatient unit. Therefore, hospitalization in a smoke-free environment is associated with increases in patients’ expectancies about quitting and staying quit.

While motivation and working on smoking cessation and reduction remain important, particularly for less motivated patients, motivation is only one aspect of quitting. It is not the only important factor for smoking cessation or reduction. This is because spontaneous and unplanned quitting are more likely to be successful for some people with mental disorder than planned quit attempts [81]. This is particularly important for people with severe mental disorder, who may have significant cognitive deficits that make reflection, planning, motivation and initiation of strategies more difficult. It highlights the important role of health professionals who can encourage immediate action. It also emphasizes the important role of environments that actively promote smoking cessation and actively discourage smoking [80]. Self-efficacy (a person’s belief in and confidence) to quit smoking or manage withdrawal has been shown to be clearly influenced by the environment, with pro-smoking environments negatively affecting self-efficacy [47,82].

### 3.6. Exposure to Other Smokers, off-Site Safety Concerns and Boredom

Where smoke-free policies do not exist, patients have reported that the culture of smoking in inpatient settings has been the direct cause of their uptake of smoking [47]. There is some evidence that a proportion of patients with mental disorder began smoking while admitted to an inpatient mental health unit [7,47]. No study has comprehensively reviewed this question; however, it is possible that up to 10% of mental health patients who smoke started while they were an inpatient. The anecdotal evidence cogently demonstrates that the smoking culture in mental health units is a catalyst for patients to take up smoking. Anecdotal evidence also demonstrates that smokers who have quit can relapse when readmitted. In a large qualitative ethnographic study of mental health patients’ experiences of care, several reported that they relapsed to smoking because “everyone else was smoking” and because there was “simply nothing else to do” [47].

Mental Health settings are also places where many patients experience significant boredom during their hospital stay due to lack of structured activities. Research has shown that lack of activity (and associated boredom) in mental health settings is associated with high levels of smoking [47,83,84,85]. The longstanding culture of smoking in mental health settings is reinforced as one of the few “normal” activities for patients within such environments. Many patients seek respite from the boredom of the unit and smoking, either alone or with other patients, is often part of that respite. This leads to staff concerns about supervision of patients during their time away from the unit. Therefore, an issue raised often in opposition to smoke-free policy is that patients who wish to continue smoking are forced to leave the hospital grounds. In many cases, this means congregating next to busy roads just outside of hospital grounds where they cannot be adequately supervised by staff. This is thought to pose a number of safety concerns, including risk of traffic accidents or attempted suicide by patients while away from the unit. However, the decision to allow a patient to leave a mental health unit is a clinical practice question and should take account of such risks. This includes risk mitigation strategies, if required. Unless directly supervised, it is not possible to control what a patient does once granted leave. Nor is risk necessarily altered by patients smoking onsite. The death of a patient usually involves collusion among a range of contributing factors that are particular to that situation. It is therefore difficult to draw broad conclusions regarding the efficacy of a policy based on individual cases, more so when the clinical circumstances of those cases vary widely. We also assert that it is the structure of treatment options that needs to be addressed, as one of the fundamental reasons why people leave the ward in the first place, whether to smoke or otherwise. This comes back to the quality of care provision.

### 3.7. Rights and Choice

A common question with regard to smoke-free policy is whether patients should retain a right to choose whether to smoke during their admission. Shattell and Andes [86] question whether smoke-free policies in mental health units are too Machiavellian, that is, whether the ends (improved long-term health) justify the means (imposing abstinence on patients who are acutely unwell). Warner [87] argues that the differential burden of stigma on smokers with mental disorder has adverse implications for their human right to dignity and choice because they are already disadvantaged. She argues that imposing smoke-free policy is a rationalist stance that ignores the complexity of the role of smoking for this population. However, the issue of choice is a complex one in mental health inpatient environments. There are many choices that are taken away from people when they enter these environments, such as what they will eat and when, sharing spaces with others who they may not wish to share spaces with, being in the environment itself, taking medications, and so on. Like tobacco, alcohol is a legal substance that has rules around its consumption and presence in these environments. Commonly, this is a prohibition; that is, it is not an accepted part of the environment under any circumstances. Tobacco consumption has historically, in part at least, been placed on the “choice” side of the fence. This is due to the role tobacco has played in the management of mental disorder as part of the cultural milieu. Treating it as a choice is, however, inconsistent with the approach applied to other activities, such as alcohol dependence [88], particularly given the fact that it is one of the greatest contributors to mortality and morbidity for these populations. The UK Royal College of Physicians’ [12] stance is that healthcare institutions have a moral imperative to promote mental and physical health, to move away from a culture that supports smoking.

Hackett [88] discussed a number of legal implications of allowing smoking in psychiatric units and stated, “while respect for patient rights and respect for patient autonomy are fundamental guiding principles of ethical medical care, the ability to smoke within a treatment setting does not qualify as a fundamental right because granting permission to smoke does not meet the principle threshold of the physician’s duty to provide competent medical care” [88]. In a Canadian study [89], staff predominantly viewed smoking as a choice and patients predominantly viewed it as an addiction, with direct adverse consequences for the level of support provided to patients because tobacco use was not framed as an addiction requiring treatment. Other studies have noted similar concerns, also noting that staff that hold that smoking is a right are more likely to oppose smoke-free policy and staff that smoke are more likely to believe in patients’ right to smoke and inability to quit [22].

### 3.8. Can Smoke-Free Policy Have Positive Impacts?

There is a significant body of evidence demonstrating that smoke-free policy has many positive benefits for the units, staff and patients concerned. In summary, the overall evidence regarding the outcome of smoke-free policy is that:

- implementation is less burdensome than staff initially fear [15,21,22,23,24,25,61,62,90,91];
- staff and patient physical wellbeing is improved [25,56,62,84];
- staff perceptions of patients’ capacity to quit smoking expand [58,62,71,92]. Longitudinal studies show that smoke-free policies bring about a change in the smoking culture, particularly shifting staff attitudes to patients’ smoking [92];
- clinical and recovery-focused care is enhanced [15,21,22,23,24,29,60,77,91,93]. Jochelson [93] found that patients were less bored and more engaged in ward activities when there was a smoke-free policy. This has been commonly reported in evaluations of smoke-free policy in drug and alcohol settings. In these settings, staff have noted significant reductions in “drug talk” post implementation and greater patient engagement in therapy programs also. Research also suggests that a smoking culture deskills staff [94] and that the imposition of smoke-free policies increase staff skillsets [23,24,71]. Lawn and Condon [94] described the dilemmas psychiatric nurses face when there are no smoking restrictions. This is not due to “lazy nursing”, but rather a demonstration of the unique ethical struggles nurses face when the culture of smoking overwhelms their attempts to provide good clinical
- nicotine dependence is addressed [15,19,90,91];
- patients’ quality of life and long-term mental health outcomes improve [43,44];
- fears about increased aggression are unfounded (they decline in some instances) [15,21,22,59,61,62,63,64]. In an Australian study in a large forensic psychiatry hospital [70], most staff felt patient care was easier with a smoke-free policy (See also [95,96]
- concerns about patients absconding and requesting discharge against medical advice are unfounded [15,21,22];
- rates of seclusion do not increase (in some instances they decrease) [15,21,22,59,63];
- staff smoking rates decline [15,23,74,90,97];
- exposure to environmental tobacco smoke declines [32,33]; and
- patients gain capacity and belief in their own ability to quit or cut down their tobacco consumption [58,63,65,71,72,77].

### 3.9. What Barriers to Smoke-Free Policy Implementation Remain to be Addressed?

Our review has identified a number of barriers to successful implementation of smoke-free policy in mental health units. A central barrier is the historical culture of smoking in these settings which has led to the perpetuation of a number of structural aspects of how care is provided. These structural aspects have then impacted on a number of practices by staff in these settings and myths held by staff about the role of smoking and their role in supporting patients’ smoking cessation. These practices and myths arise essentially as a result of problems with staff knowledge, skills and values that need to be addressed within the culture of service provision to people with mental disorder. They may improve as recovery-based philosophies of care become the norm within this field [98]. Evidence of psychiatric staff being slow to recognise and act regarding smoking and mental disorder is demonstrated by several studies [21,22,47,85,99,100,101]. Research demonstrates that staff often present with greater resistance to smoke-free policies than the patient population [15,22,28,58,63,99,100]. This may be due, in part, to a higher proportion of staff working in mental health facilities being smokers compared to staff in other healthcare contexts. It is especially pertinent to psychiatric nurses because they have higher rates of smoking than other staff and they also provide the bulk of direct care to patients [11,94,100]. Several studies have highlighted the importance of staff training for the success of smoke-free policies [15,16,22,60,62,89,90,91,92,99,100,101]. This includes training in the effective use of NRT, recognition and management of nicotine withdrawal, medication management, policy enforcement and dealing with violations, and training which help to address cultural issues. One means to address this problem is for university programs for undergraduates and post-graduate health professionals to ensure that smoking and mental disorder is a core component of learning.

However, our review has also highlighted the complex array of concerns that arise within these settings. It suggests that solutions are not always as straightforward as declaring the intention to implement the policy, inform and educate those concerned, and expect the path to be smooth. These settings continue to be a “barren wasteland of boredom” for many patients, regardless of whether there is a smoke-free policy in place or not, because of the scarceness of structured activities for all patients. Rather, a multi-pronged and integrated approach across inpatient and community settings that addresses all of these concerns and more is likely to be needed.

### 3.10. What Makes Smoke-Free Policy Implementation Successful?

Various factors have been shown to be beneficial for the successful implementation of smoke-free policies. These include adequate consultation with staff and patients to alleviate their fears, sufficient staff training, supporting staff to quit smoking or abstain while at work, clear leadership and management support, clear audit and reporting of all patients’ smoking status, adequate resourcing of policy implementation, close follow-up of patients after discharge, support across the continuum of care transitions, and consistent implementation practices in mental health inpatient units [15,18,23,25,26,55,58,70,102,103]. These factors form part of key recommendations for effective implementation of smoke-free policy in mental health settings, arising from the international research evidence. More specifically, this evidence recommends consulting staff and patients, in order to provide opportunities for open and frank discussion and collaborative problem-solving about how to proceed, to hear people’s concerns, and to provide opportunities for regular feedback. Further recommendations include dedicated staff resources to provide ongoing mentorship and support to staff teams implementing the policy and provision of training for all staff. Audits of smoking cessation processes that include collection of clearly defined and evidence-based performance indicators are recommended. Finally, recommendations arising from the international evidence also include routine and ongoing incorporation of nicotine withdrawal management into clinical care, and management of “high risk” smokers by smoking cessation specialists.

### 3.11. Limitations

The literature reviewed here was examined with a particular lens, defined by the paper’s objectives. We acknowledge our potential for bias as researchers and clinicians who have a clear pro smoke-free policy stance. We did not undertake a systematic review, nor did we examine all aspects of previous research. Rather, we aimed to identify specific enablers and barriers to successful implementation. Also, this review was biased towards Australian, UK and US studies. Therefore, we refer the reader to the NICE guidance series [19,20,21,22] in this area for a more comprehensive review. Only one study with veteran psychiatric populations was included and, given there may be unique cultural considerations for this population, we do not purport to cover the needs of this population. There were also a small number of forensic psychiatry studies. Finally, we did not focus on smoking cessation strategies or community mental health settings and their implementation of smoke-free policy.

## 4. Conclusions

It is important to consider that, like many policies, smoke-free policy implementation is a process, not an event. Evidence of problems does not mean the policy is inappropriate or a failure. Addressing the damage caused by tobacco for people with mental disorder requires a multipronged approach across the continuum of care. It involves hospital and community services, to ensure initiation of smoking and relapse to smoking within pro-smoking cultural environments do not occur [19,20,21,22]. This is also important, given that many people with mental disorder do not access inpatient care for their mental health needs [104]. It is also important given that many people with mental disorder do not receive adequate mental health care. For example, while only 10% of people with mental disorder in Europe receive notionally adequate treatment [105], the majority with mental disorder receive treatment from primary care where their smoking is usually neglected.

Concentrating smoking cessation efforts in the community is pointless if patients re-enter impatient settings when unwell and relapse with their smoking due to culture of smoking in those environments. Smoke-free policy in inpatient mental health units needs to complement community based smoking cessation programs. The adverse effects associated with smoke-free policy in mental health settings can be ascribed to the way in which the policy has been implemented, rather than the policy itself [18]. In particular, partial smoke-free policies have been shown to be particularly problematic. The culture of smoking by patients in inpatient psychiatric hospitals needs to be challenged by asking questions such as “Why is smoking perceived as their only pleasure, how did it get to be perceived as such, and what responsibility do services have to address this?” [35]. Smoke-free policy in inpatient mental health settings is one of the most important means of addressing the culture of smoking and answering these questions.
